# Repeated Intrathecal Stem Cells Optimize Neuroplasticity and Motor Function in Moderate‐to‐Severe Cerebral Palsy of Rats

**DOI:** 10.1155/sci/4337435

**Published:** 2025-12-28

**Authors:** Yi Xu, Ting–Ting Peng, Shiya Huang, Xiaolin Guo, Jie Luo, Tingting Peng, Liru Liu, Mingshan Han, Ting Gao, Hongmei Tang, Jing Zhang, Lu He, Kaishou Xu

**Affiliations:** ^1^ Department of Rehabilitation, Guangzhou Women and Children’s Medical Center, Guangzhou Medical University, Guangzhou, Guangdong, China, gzhmc.edu.cn; ^2^ Department of Sports and Health, Guangzhou Sport University, Guangzhou, Guangdong, China, gipe.edu.cn; ^3^ School of Exercise and Health, Shanghai University of Sport, Shanghai, China, sus.edu.cn

**Keywords:** animal model, cerebral palsy, intrathecal infusion, mesenchymal stem cell, repeated injections

## Abstract

Human umbilical cord‐derived mesenchymal stem cells (hUC‐MSCs) ameliorate motor deficits in cerebral palsy (CP), but the effect of injection frequency remains unclear. Moreover, most studies have focused on mild CP models (unilateral carotid artery occlusion [UCAO] model). This study explored the effect and mechanism of hUC‐MSCs in a rat model of moderate‐to‐severe CP (bilateral carotid artery occlusion [BCAO] model). On postnatal Day 4 (P4), Wistar rat pups underwent BCAO induction. Subsequently, they received either a single intrathecal injection of hUC‐MSCs on P21 or repeated injections on P21, P28, P35, and P42. Motor performance was assessed using the rotarod and front‐limb suspension tests, while neuronal regeneration and inflammation were evaluated via biomarkers including neuronal nuclear antigen (NeuN), ionized calcium‐binding adapter molecule‐1 (Iba‐1), glial fibrillary acidic protein (GFAP), myelin basic protein (MBP), and brain‐derived neurotrophic factor (BDNF). P18 model screening confirmed that the BCAO model resulted in more severe brain damage and motor impairment than the UCAO model. After injection of lentivirally transfected hUC‐MSCs, it was found that hUC‐MSCs could nest in the damaged area and survive for at least 3 days. Administration of hUC‐MSCs following BCAO modeling led to notable improvements in both behavioral performance and histological outcomes. Furthermore, repeated injections offered greater therapeutic benefits compared to single injection. It indicated that the efficacy of repeated injections of hUC‐MSCs in the treatment of moderate‐to‐severe CP was superior to that of single injection. Its mechanism was related to the improvement of damaged myelin structure, reduced immunoinflammatory responses, and increased neurotrophic support.

## 1. Introduction

Cerebral palsy (CP) is a lifelong condition that often manifests as motor dysfunction, imposing significant burdens on affected individuals, their families, and society at large [1–3]. Conventional rehabilitation techniques are primarily symptomatic, aiming to maximize children’s functional performance and task skills, thus to improve their self‐care ability and quality of life. However, due to the unmet need for brain injury repair, the therapeutic efficacy is limited [4], prompting a continuous search for new therapeutic options.

Recent findings suggest that stem cell transplantation may be a potential new approach targeting brain injury. Unlike other types of stem cells that migrate and replace damaged cells, mesenchymal stem cells (MSCs) exert their therapeutic effects predominantly through paracrine signaling and immunomodulation, offering a safer and more efficient approach [5]. In addition, the MSCs, which exhibit advantageous properties, including low tumorigenicity, low immunogenicity, and low heterogeneity, have made them widely utilized in scientific research and clinical applications [6, 7]. A meta‐analysis of clinical studies endorsed the safety and efficacy of stem cell treatment for motor dysfunction in CP and recommended human umbilical cord‐derived MSCs (hUC‐MSCs) as a promising intervention [8]. Meanwhile, Amanat et al. [9] found that the diffusion tensor imaging (DTI) of children with CP indicated the alterations of white matter integrity following a single injection of hUC‐MSCs, objectively demonstrating the great therapeutic potential of stem cells. However, there is a lack of scientific and standardized guidance for the formulation of clinical protocols for stem cell transplantation. Variability in injection schedules, transplantation routes, and dosage regimens complicates the establishment of uniform clinical recommendations [10, 11]. Current research predominantly focuses on dosing, with evidence indicating that higher doses are more effective than lower ones [12, 13]. Nevertheless, administering too many cells in a single injection carries the risk of embolization [14, 15]. A small number of repeated injections may be an effective solution to this issue [16, 17], although the efficacy remains unclear.

In addition, most studies related to stem cell intervention in CP have focused on hemiplegic CP. The Rice–Vannucci rat model (unilateral carotid artery occlusion [UCAO]) is the earliest and most well‐researched neonatal model of hypoxic‐ischemic encephalopathy, which is frequently employed to simulate hemiplegic CP [18, 19]. Nonetheless, higher proportions of bilateral motor dysfunction caused by extensive brain lesions are observed in clinic. This discrepancy highlights the limitations of the UCAO model [20]. It has been demonstrated that the brains of rats subjected to bilateral carotid artery occlusion (BCAO) in the early stages of brain development exhibit periventricular leukomalacia (PVL), which corresponds with the clinical imaging characteristics observed in children with moderate‐to‐severe CP [21, 22]. Moderate‐to‐severe CP is accompanied by severer motor dysfunction. Effective, clinically targeted treatments for moderate‐to‐severe CP remain elusive, and preclinical research in this area is limited [23, 24]. An animal model that adequately simulates bi‐hemispheric involvement in children with moderate‐to‐severe CP is crucial for developing therapeutic interventions. Therefore, this study established both UCAO and BCAO model to compare the severity of the resulting brain injuries and motor dysfunction.

Up to now, few studies have assessed the effect of hUC‐MSCs on moderate‐to‐severe CP, and the efficacy remains unclear. In our previous research, we investigated the impact of transplantation route on ameliorating motor dysfunction in rats with hemiplegic CP [25]. We recommend intrathecal injection as the optimal route for stem cell transplantation. This study first investigated whether BCAO could serve as a moderate‐to‐severe CP model and then focused on determining whether behavioral improvements and mechanisms (remyelination, immunomodulation, and neurotrophic regulation) mediated by repeated intrathecal hUC‐MSC administration.

## 2. Materials and Methods

### 2.1. Cells

The second generation of hUC‐MSCs was sourced from Saliai (Guangzhou, China). The hUC‐MSCs were cultured following the manufacturer’s protocols. Briefly, the hUC‐MSCs were cultured in the serum‐free specialized medium for human MSCs (Saliai, Guangzhou, China) supplemented with 1% penicillin–streptomycin solution at 37°C in a 5% CO_2_ and 95% air incubator. Adherent hUC‐MSCs were digested with trypsin for 2 min, collected, washed twice with PBS, and prepared as a single‐cell suspension. Subsequently, cells were mixed with antibodies and incubated at room temperature in darkness for 15 min, followed by detection and analysis via flow cytometry.

The hUC‐MSCs were transfected with lentivirus encoding red fluorescent genes to achieve stable fluorescence expression. The lentivirus was obtained from HanBio Technology (Shanghai, China). Its vector information is pHBLV‐EF1‐MCS‐CMV Uro, the target gene sequence is MTS‐turboRFP, and the titer is 2 × 10^8^. Then, the hUC‐MSCs were inoculated into a six‐well plate at a density of 2 × 10^5^ cells/well. When the cells were 60% confluent, they were infected at a multiplicity of infection (MOI) of 20. After 48 h of infection, 3 μg/mL puromycin was added to the culture medium to screen for stably transfected cells. The medium was replaced every 48 h until uninfected cells completely perished.

### 2.2. Animal Procedures

All experimental procedures were approved by the Experimental Animal Ethics Committee of Guangzhou Medical University (Acceptance Number: G2020‐257). Rats were housed under standard conditions, maintained in a quiet environment with controlled temperature (22–25°C) and humidity (40%–70%), with a 12 h light/dark cycle, and provided with a standard laboratory diet and water. Thirteen SPF Wistar pregnant rats were obtained from Liaoning Changsheng Biotechnology (NO. SCXK Liao2020‐0001, Liaoning, China), and 135 pups were born in 13 litters.

All modeling was conducted under isoflurane anesthesia (induction dose 4%, surgical dose 1.5%~2%). The UCAO model achieved ischemia by ligation of the left common carotid artery of P7 rats (*n* = 24), which were subsequently placed in 37°C, 8% oxygen, and 92% nitrogen incubator for 3 h to induce hypoxia. In the BCAO model, bilateral common carotid arteries of P4 rats (*n* = 87) were occluded, followed by 15 min in the hypoxic chamber under the same conditions. All pups that underwent UCAO surgery survived (*n* = 24). Five pups died within a week after BCAO surgery (*n* = 82). Rats undergoing modeling were scheduled for model screening at P21. Motor function was assessed using the rotarod test and front‐limb suspension test. Additionally, growth and brain development were assessed through weight measurement, Nissl staining, and magnetic resonance imaging (MRI).

**Figure 1:**
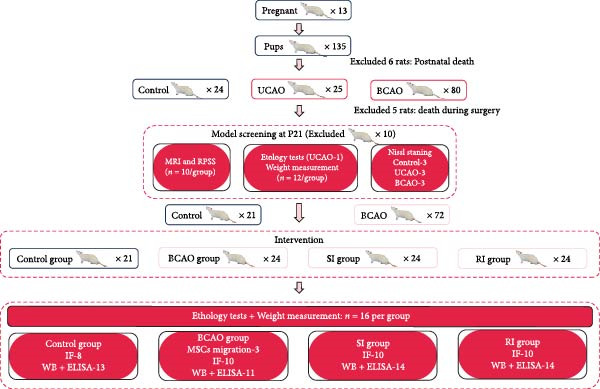
Experimental group distribution. BCAO, bilateral carotid artery occlusion model; ELISA, enzyme‐linked immunosorbent assay; IF, immunofluorescence; MRI, magnetic resonance imaging; RPSS, rat pup severity score; RI, repeat injection; SI, single injection; UCAO, unilateral carotid artery occlusion model; WB, Western blot.

This study consisted of five groups. The control group did not undergo surgery or any intervention (*n* = 24). The UCAO group underwent UCAO surgery but did not receive any intervention (*n* = 24). The BCAO group, SI group (single injection group), and RI group (repeat injection group) all underwent BCAO surgery. The difference was that the BCAO group did not receive any intervention (*n* = 24), the SI group received 1 × 10^6^ hUC‐MSC intrathecally at P21 (*n* = 24), and the RI group received 1 × 10^6^ hUC‐MSC intrathecally at P21, P28, P35, and P42 (*n* = 24) [25–28]. These rats were euthanized at either P35 or P49 for tissue sampling. Blinding was fully implemented for all behavioral tests (by independent operators), molecular assays (using coded samples), and data analysis (by independent operators). The rat assignment of the whole experiment is shown in Figure 1.

### 2.3. Ethology Tests

The rotarod test and front‐limb suspension test were performed at P20 to assess motor coordination and forelimb strength among the groups.

In the rotarod test, rats were placed on a rotating rod with a diameter of 5 cm and allowed to adapt to a speed of 4.0 r/min for 5 min. Then, the apparatus was set to a uniform acceleration mode from 4.0 r/min to 40 r/min, the time from the start until the rat fell was recorded, and the average of three measurements was taken.

For the front‐limb suspension test, a wire with a diameter of 0.5 cm and a length of 40 cm was placed 50 cm above the experimental platform, and a buffer pad was placed below. After grasping the steel wire, it was released with its hind limbs suspended. Record the time from release to fall of rats, measure three times, and take the average value.

### 2.4. Weight Measurement

The weight development of the rats was recorded. Weight measurements began 12 h after the pups’ birth and were subsequently conducted every 7 days. The average of three weight measurements was taken at each time point.

### 2.5. MRI Detection and Rat Pup Severity Score (RPSS) Analysis

Following intraperitoneal injection of sodium phenobarbital for anesthesia in rats, they were positioned prone and placed into an MRI scanner (Sigma Prime 3.0 T, Germany) for continuous coronal scanning of 30 slices. The parameters were set to a voxel size of 0.3 mm × 0.3 mm × 0.9 mm, with T2‐weighted imaging (TE = 103 ms, TR = 4390 ms).

To objectively correlate neuroimaging findings with motor dysfunction severity, we employed the RPSS based on T2‐weighted MRI scans. In brief, scoring was based on the MRI scan slice results from anterior to posterior slices. The observation area includes the dorsal and ventral cortex, subcortical regions (basal ganglia and thalamus), and hippocampus. Each slice was divided into four quadrants: abnormal signals within a quadrant were scored 1 point, while normal signals received 0 points. The final RPSS result was calculated by summing the slice scores and dividing by the total number of slices.

### 2.6. Nissl Staining

Rats were euthanized and subsequently subjected to intracardiac perfusion with PBS and 4% paraformaldehyde. Following perfusion, brain tissue was isolated and processed into paraffin sections according to standard procedures. In paraffin sections, the brain tissue was placed in a drying oven and heated continuously at 67°C for 60 min, followed by immersion in xylene for ~40 min for dewaxing. Nissl staining solution was applied at 57°C for 30 min. The slices were washed continuously with double‐distilled water until the stain faded, followed by sequential immersion in 95% ethanol for 50 s, anhydrous ethanol for 5 min, and xylene for 10 min. Finally, the stained sections were observed under a microscope.

### 2.7. Western Blot (WB)

After euthanasia and cardiac perfusion with PBS, the M1 region of the motor cortex was isolated from P49 rat brain tissue. The tissue was fully lysed with an ultrasonic crusher, and the protein concentration was measured with the bicinchoninic acid (BCA) method. The protein concentration of the sample was then adjusted to achieve a final concentration of 5 μg/μL. The proteins were separated by sodium dodecyl sulfate‐polyacrylamide gel electrophoresis and transferred to a polyvinylidene fluoride membrane. Then, the membrane was incubated with rabbit anti‐neuronal nuclear antigen (NeuN Absolute, A19086,1:2000), rabbit anti‐myelin basic protein (MBP; CST, 78896S, 1:1000), rabbit anti‐glial fibrillary acidic protein (GFAP; Protein, 16825‐1‐AP, 1:2000), and rabbit anti‐brain‐derived neurotrophic factor (BDNF; Absolute, A18129, 1:2000) to detect the content of the target protein. Glyceraldehyde 3‐phosphate dehydrogenase (GAPDH) was used as an internal reference to quantitatively detect the target protein, and the detection results were analyzed using imageJ.

### 2.8. Immunofluorescence (IF)

The rats were euthanized and perfused, after which the whole brain was extracted. The brain tissue was fixed overnight with 4% paraformaldehyde in a 4°C refrigerator. The brain tissue was then dehydrated in 25% sucrose for 48 h, embedded in optimal cutting temperature (OCT) compound, and stored at −80°C until use. Cut the embedded tissue into 12 μm slices on a coronal plane. Frozen sections were washed and permeabilized with TBS and TBST and then blocked with 5% goat serum at room temperature for about 1 h. Subsequently, the sections were incubated overnight with primary antibodies: rabbit anti‐ionized calcium‐binding adapter molecule‐1 (Iba‐1; Abcam, ab153696, 1:200), rabbit anti‐NeuN (Absolute, A19086,1:200), rabbit anti‐MBP (CST, 78896S, 1:50), rabbit anti‐GFAP (Protein, 16825‐1‐AP, 1:200), and rabbit anti‐BDNF (Absolute, A18129, 1:200) at 4 °C. After rinsing, the slices were incubated with goat anti‐rabbit IgG H&L (Alexa Fluor 594/488, Abcam, ab150080, 1:500) at room temperature for 1 h. The cell nuclei were stained with DAPI solution for 8 min and the sections were mounted with anti‐fade mounting medium. Finally, obtain IF images under a fluorescence microscope and the fluorescence intensity or cell number was quantified using ImageJ.

### 2.9. Enzyme‐Linked Immunosorbent Assay (ELISA)

After removal of the rat brain, an appropriate amount of tissue from the M1 area of the motor cortex was weighed and placed on ice. The tissue was homogenized using ultrasound in precooled PBS supplemented with protease inhibitors (100:1), followed by centrifugation to collect the supernatant. According to the manufacturer’s protocol, the expression levels of interleukin‐6 (IL‐6) and tumor necrosis factor alpha (TNF‐*α*) were detected using an ELISA kit. Measurements were taken from three standard samples and all experimental samples with a 96‐well plate.

### 2.10. Statistical Analysis

The experimental data were analyzed using IBM SPSS Statistics 25, and all data were presented as mean ± SEM. Comparisons between groups were conducted using analysis of variance or nonparametric rank sum tests to determine significance, followed by post hoc analysis. If the data conformed to a normal distribution and had homogeneous variances, a one‐way ANOVA was used; otherwise, nonparametric tests (e.g., Kruskal‐Wallis test) were used. *p* < 0.05 indicates that the difference is statistically significant. Use GraphPad Prism 9.5 to display the experimental results.

## 3. Results

### 3.1. Moderate‐to‐Severe CP Model was Successfully Established

To determine the effect of brain injury severity on growth and brain development in rats, the model was screened on day P20. The results of weight measurement (P0~P21) showed no significant differences in weight between the UCAO group and the Control group (*p* > 0.05) after surgery. In contrast, the body weight of the BCAO group was significantly lower than that of the other two groups (*p* < 0.01, Figure 2A). The results of the rotarod and front‐limb suspension tests are shown in Figure 2B. Compared with the rotation and front‐limb suspension time of the Control group (221.91 ± 22.56 s, 210.78 ± 20.45 s), the UCAO group (137.44 ± 11.29 s and 137.22 ± 15.36 s, *p* < 0.01), and the BCAO group (114.50 ± 15.77 s and 88.00 ± 18.32 s, *p* < 0.01) both showed a significant decrease. In addition, the BCAO group showed shorter times than the UCAO group in both tests (*p* < 0.01, for both), indicating more severe motor dysfunction in BCAO rats.

Figure 2Successfully established moderate‐to‐severe cerebral palsy rat models: (A) Changes in body weight of rats in each group from P0 to P21 (*n* = 12, per group). (B) Changes in motor function of rats in each group at P21 (*n* = 12, per group). (C) Nissl staining results of M1 area in the motor cortex. (D) Analysis of the number of neurons (*n* = 3, per group). (E) Three consecutive magnetic resonance imaging scan results from front to back for each group. (F) Rat pup severity score based on magnetic resonance imaging detection results (*n* = 10, per group). ^▲^: vs. Control group, *p* < 0.05; ^#^: vs. UCAO group, *p* < 0.05. g, gram; s, second.

**Figure:**
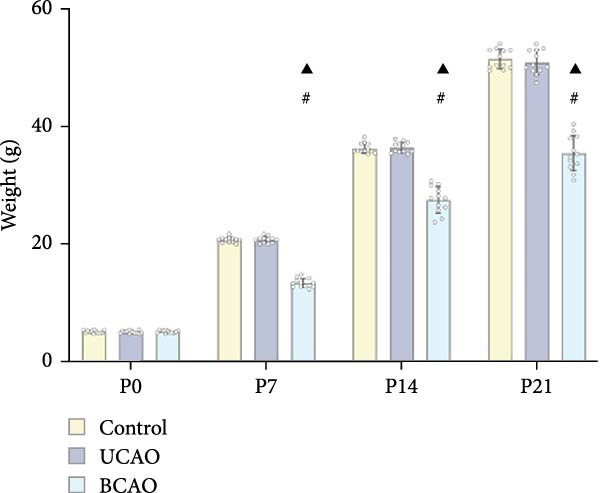
(A)

**Figure:**
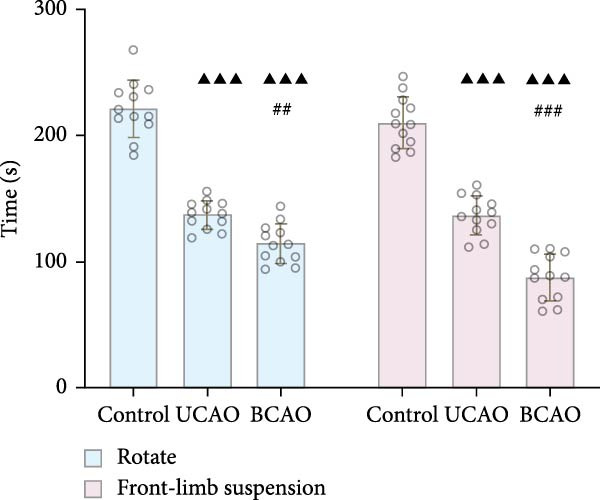
(B)

**Figure:**
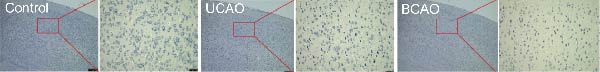
(C)

**Figure:**
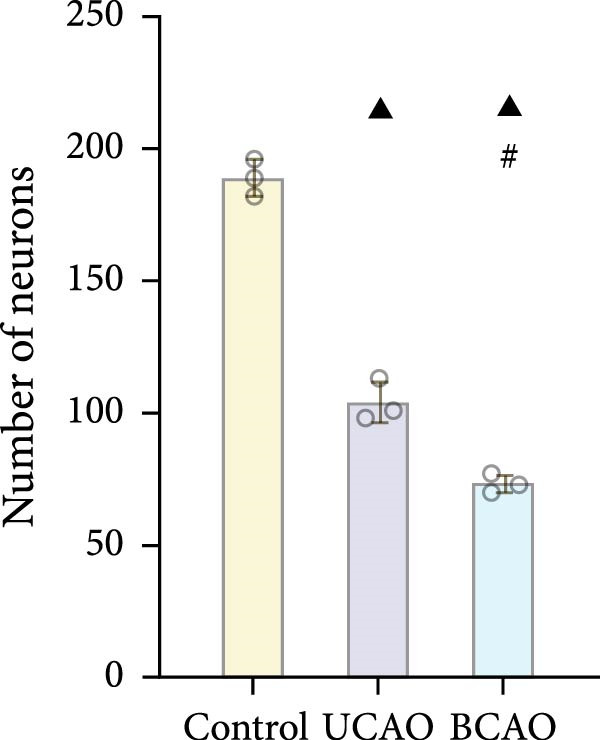
(D)

**Figure:**
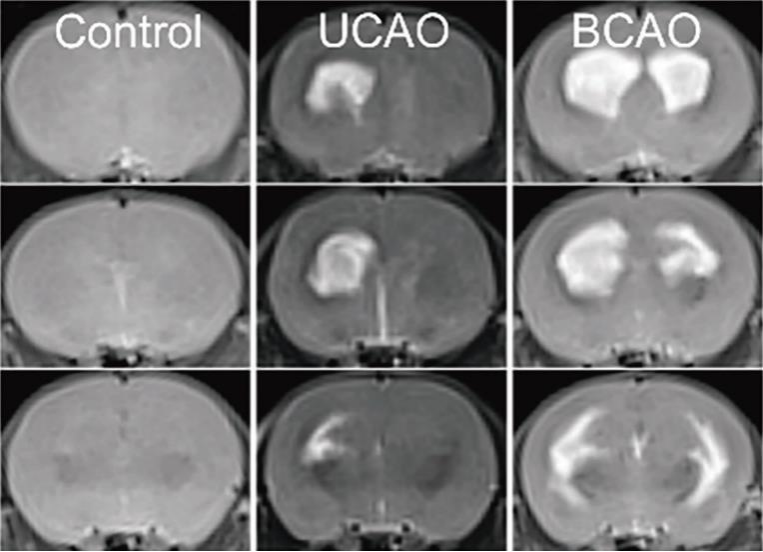
(E)

**Figure:**
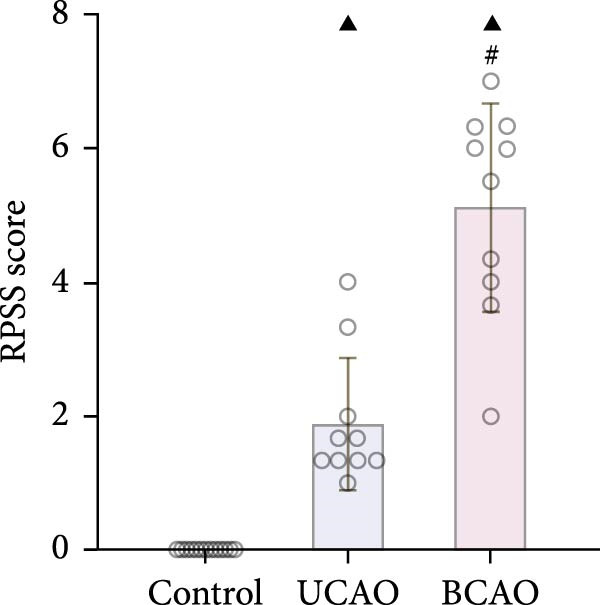
(F)

Subsequently, MRI and Nissl staining were employed to assess the severity of brain injury in rats. The Nissl staining results of the primary motor cortex (M1) area of the brain tissue slices showed that both the UCAO group and the BCAO group exhibited smaller cell bodies, a sparser arrangement, and fewer neurons than the Control group (*p* < 0.01, Figure 2C,D). Moreover, the BCAO group had more severe damage than the UCAO group (*p* < 0.01, Figure 2C,D). The MRI results indicated that the injury areas in the UCAO group were limited to the left hemisphere, while bilateral injuries were present in the BCAO group (Figure 2E). RPSS analysis showed that the damage levels of the UCAO group were mild (*n* = 8) and moderate (*n* = 2), while those in the BCAO group were moderate (*n* = 4) and severe (*n* = 6, Figure 2F). These findings indicate that the BCAO model, representing moderate‐to‐severe CP, was successfully established.

### 3.2. Repeated Injection of hUC‐MSCs Migrated to Brain‐Damaged Areas and Survived for More Than 3 Days

According to the standards established by the International Society for Cell Therapy, CD44, CD73, and CD105 were used as positive markers for MSCs, while CD34, and CD45 were served as negative markers [29]. The expression of each antibody was consistent with the standards for MSCs (Figure 3A).

Figure 3Transfection effect of stem cells and influence of intrathecal injection of stem cells on body weight and behavior. (A) Flow cytometry detection results of mesenchymal stem cells (positive markers CD73, CD105, and CD44; negative markers CD34 and CD45). (B) Transfection and enrichment results of mesenchymal stem cells. (C) Migration results of stem cells after 3 days of injection (*n* = 3). (D) Injection timeline. (E) Weight development between different groups (*n* = 12, per group). (F) Comparison of motor function between groups (*n* = 16, per group). ^▲^: vs. Control group, *p* < 0.05;  ^∗^: vs. BCAO group, *p* < 0.05; ^■^: vs. SI group, *p* < 0.05. APC, allophycocyanin; BV421, brilliant violet 421; CD34, hematopoietic progenitor cell antigen CD34; CD44, cluster of differentiation 44; CD45, protein tyrosine phosphatase receptor type C; CD73, Ecto‐5′‐nucleotidase; CD105, endoglin; DAPI, 4′,6‐diamidino‐2‐phenylindole; FITC, fluorescein isothiocyanate; PE, phycoerythrin; RFP , red fluorescent protein.

**Figure:**
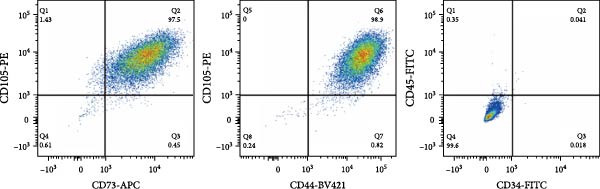
(A)

**Figure:**
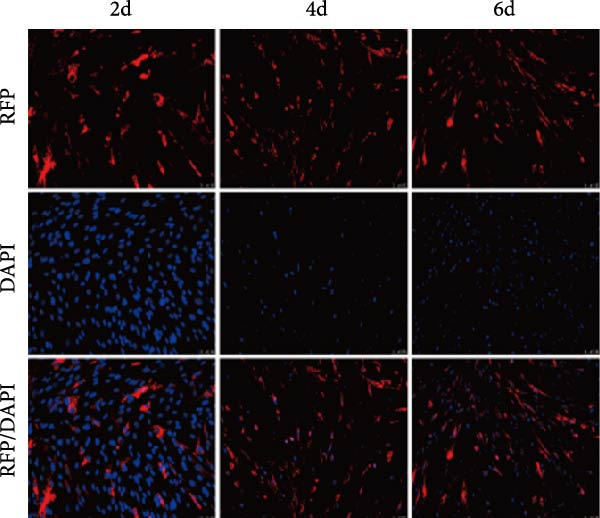
(B)

**Figure:**
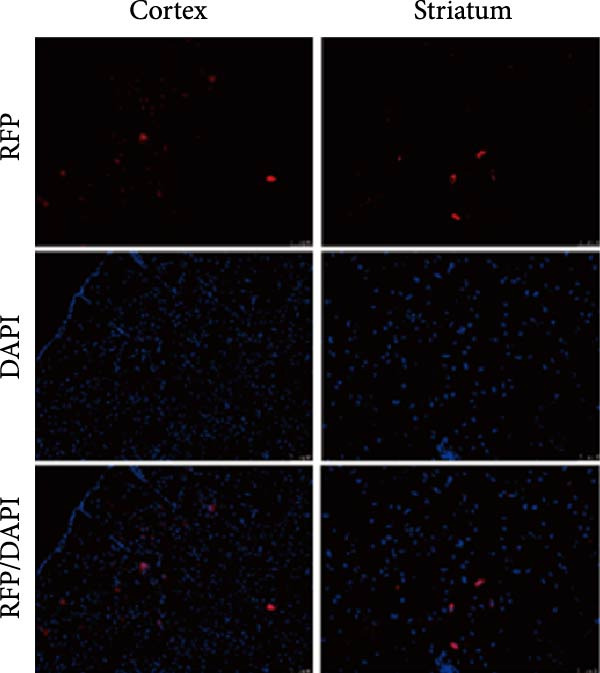
(C)

**Figure:**
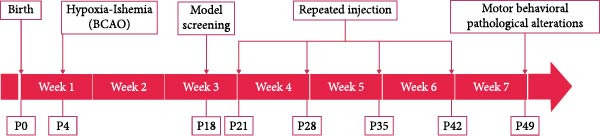
(D)

**Figure:**
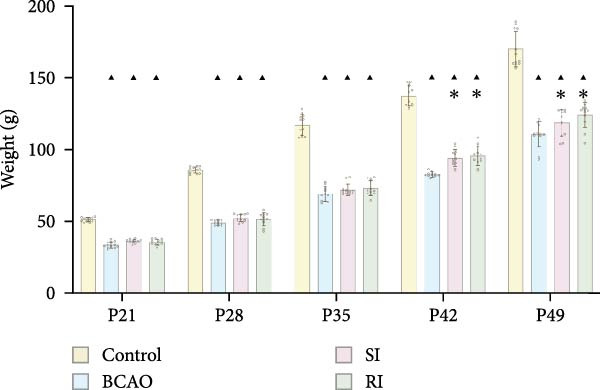
(E)

**Figure:**
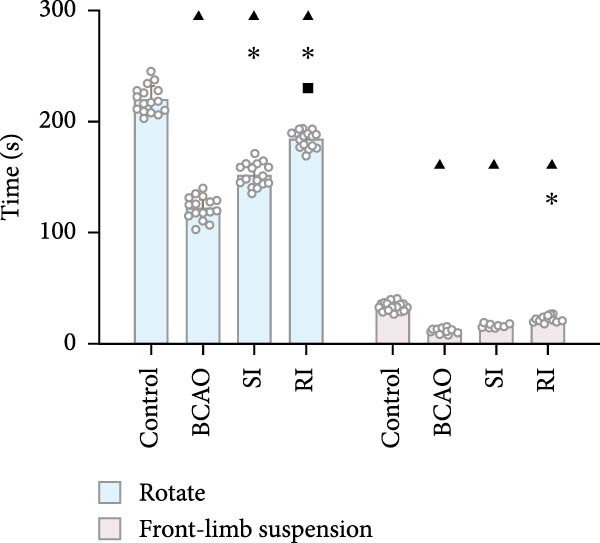
(F)

MSCs could migrate to the site of injury, secrete relevant chemokines, cytokines, and growth factors to exert therapeutic effects. In this study, we tracked MSC migration by lentiviral transfection. Approximately 98% of the cells contained red fluorescent protein after screening the transfected hUC‐MSCs with puromycin for 4–6 days (Figure 3B), indicating the successful construction of a stable cell line carrying red fluorescent protein. The stem cells above were intrathecally injected into BCAO rats. These rats were sacrificed on Days 3 and 7 after injection, and the brains were sectioned for fluorescence detection. On day 3 after injection, fluorescent signals were observed in the cerebral cortex and striatum (Figure 3C). The timeline of stem cell injections is summarized in Figure 3D. The results indicate that intrathecally injected hUC‐MSCs successfully migrated to the brain injury area and survived for more than 3 days.

### 3.3. Repeated Injection of hUC‐MSCs Improved Motor Function

As shown in Figure 3E, the weight of the Control group (*p* < 0.001) from P21 to P49 was significantly higher than that of the other groups, with no significant differences observed among the BCAO group, SI group, and RI group (*p* > 0.05). These results indicate that stem cell treatment does not influence the weight development of rats with moderate‐to‐severe CP.

To evaluate the improvement of motor function, the rotarod and front‐limb suspension tests were conducted on each group at P49. The results showed that at P49 (Figure 3F), the rotarod latency and suspension time of the BCAO group (11.58 ± 2.08 s, 122.29 ± 10.33 s) were the shortest. The RI group (21.86 ± 2.76 s, 184.21 ± 7.52 s) demonstrated greater improvements compared to the SI group (16.15 ± 1.43 s, 151.98 ± 10.28 s, *p* < 0.01), indicating that repeated injection could significantly improve the motor function of BCAO rats.

### 3.4. Repeated Injection of hUC‐MSCs Increased the Number of Neurons and the Content of Myelin Sheath

Compared with the BCAO group, the neuronal content in both the SI and RI groups increased (Figure 4A,C–E, *p* < 0.01), and the improvement in the RI group was more significant than that in the SI group (Figure 4A,C–E, *p* < 0.05). The results of myelin staining showed consistent changes in the M1 and corpus callosum (CC) regions (Figure 4D,F,G, *p* < 0.01). Although the expression level of MBP in the RI group was higher than that of the SI group (Figure 4B, *p* < 0.05), it remained lower than that of the control group (Figure 4B, *p* < 0.05). These results indicate that repeated injections increase neuronal count and myelin sheath content in the M1 and CC regions.

Figure 4Recovery trend of myelin wrapping in neurons and their axons. (A–C) Western blotting images of neuronal nuclear antigen (*n* = 6, per group), myelin basic protein (*n* = 5, per group), and glyceraldehyde 3‐phosphate dehydrogenase and the quantitative analysis graph. (D) Immunofluorescence staining results of neuronal nuclear antigen and myelin basic protein in primary motor cortex and corpus callosum regions. (E) Analysis of immunofluorescence results of neuronal nuclear antigen (*n* = 8, per group). (F,G) Immunofluorescence analysis results of primary motor cortex and corpus callosum regions (*n* = 8, per group). ^▲^: vs. Control group, *p* < 0.05;  ^∗^: vs. BCAO group, *p* < 0.05; ^■^: vs. SI group, *p* < 0.05. CC, corpus callosum; GAPDH, glyceraldehyde 3‐phosphate dehydrogenase; M1, primary motor cortex.

**Figure:**
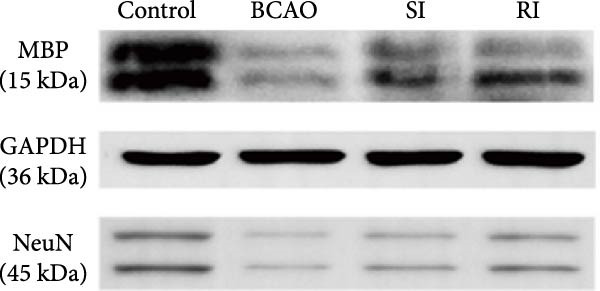
(A)

**Figure:**
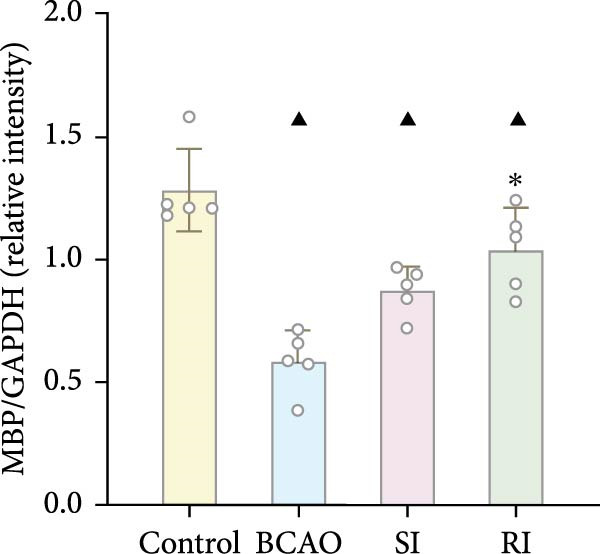
(B)

**Figure:**
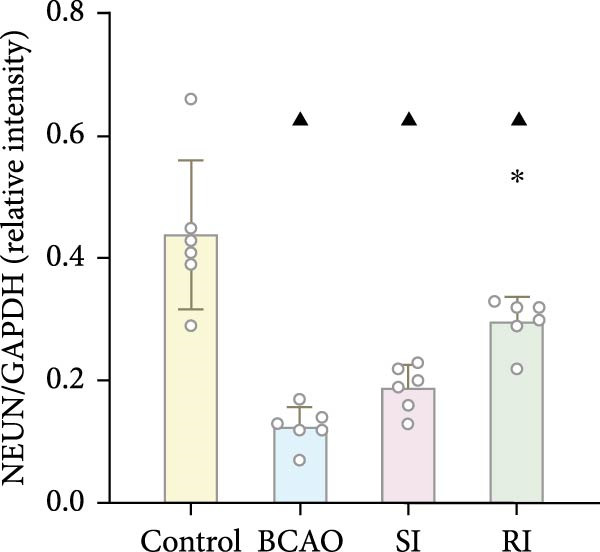
(C)

**Figure:**
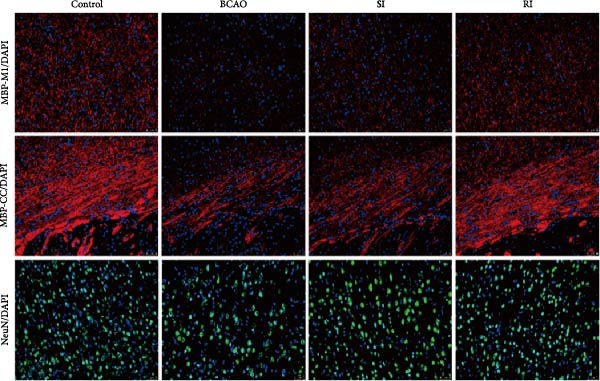
(D)

**Figure:**
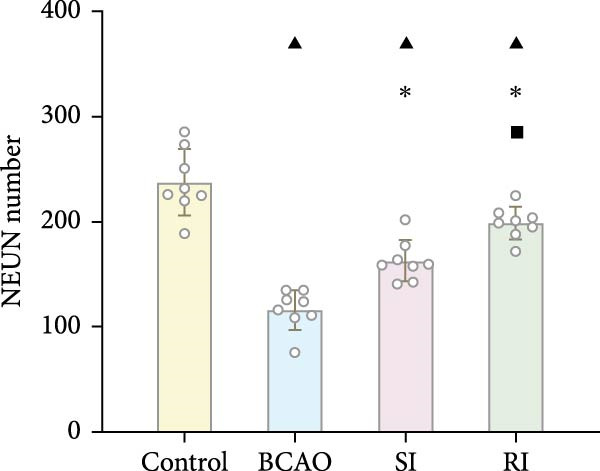
(E)

**Figure:**
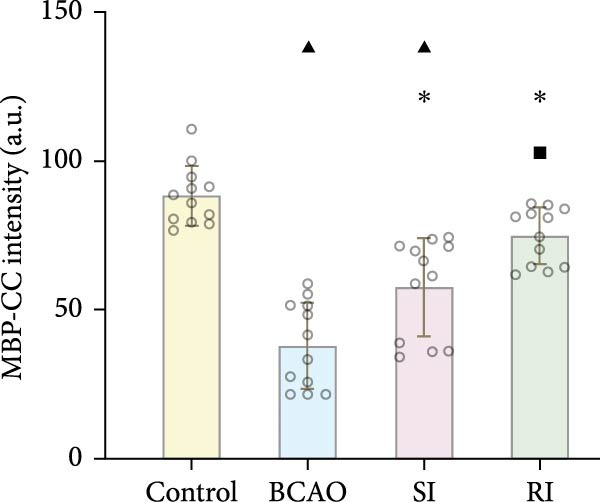
(F)

**Figure:**
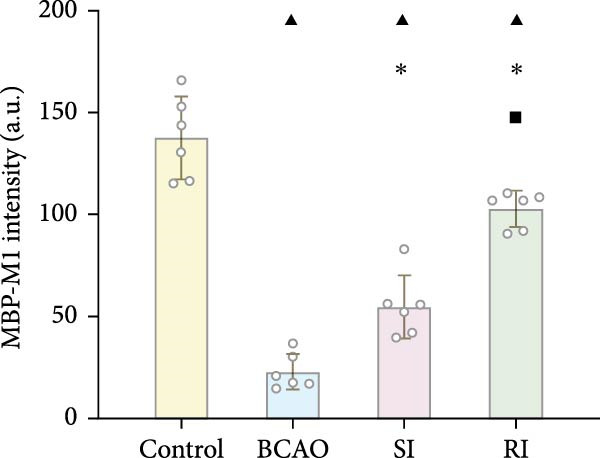
(G)

### 3.5. Repeated Injection of hUC‐MSCs Promoted Further Reduction of Inflammatory Response in the Brain

Because CP is associated with persistent inflammation, we investigated whether hUC‐MSC treatment affects the number of astrocytes and microglia. The hUC‐MSC treatment significantly reduced the expression levels of GFAP and Iba‐1 in both the SI and RI groups compared to the BCAO group (Figure 5A–E, *p* < 0.05). In further comparisons, the expression level of Iba‐1 in the RI group was lower than that in the SI group (Figure 5E, *p* < 0.01). Although the expression level of GFAP in the RI group was higher than that in the SI group, the difference was not statistically significant (Figure 5B,C). Compared to the BCAO group, the expression levels of TNF‐*α* and IL‐6 in the RI group were significantly decreased (Figure 5F,G, *p* < 0.01), while the SI group did not show a significant reduction.

Figure 5Analysis of glial fibrillary acidic protein, ionized calcium‐binding adapter molecule‐1 mediated inflammation and brain‐derived neurotrophic factor after intervention. (A,B) Western blotting images and quantitative analysis of glial fibrillary acidic protein and glyceraldehyde 3‐phosphate dehydrogenase (*n* = 6, per group). (C–E) Analysis of immunofluorescence results of glial fibrillary acidic protein and ionized calcium‐binding adapter molecule‐1 (*n* = 6, per group). (F,G) Analysis of concentration results of interleukin‐ 6 and tumor necrosis factor alpha in the motor cortex of rats in each group based on enzyme‐linked immunosorbent assay (*n* = 6, per group). (H,I) Western blotting images and quantitative analysis of brain‐derived neurotrophic factor among groups (*n* = 6, per group). (J,K) Immunofluorescence results and quantitative analysis of brain‐derived neurotrophic factor between groups (*n* = 6, per group). ^▲^: vs. Control group, *p* < 0.05;  ^∗^: vs. BCAO group, *p* < 0.05; ^■^: vs. SI group, *p* < 0.05. a.u., absorbance unit; ml, milliliter; pg, picogram.

**Figure:**
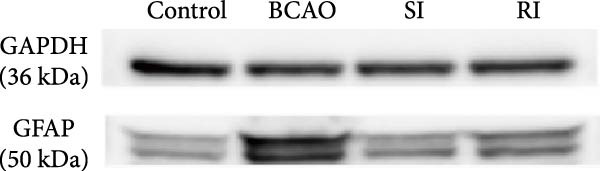
(A)

**Figure:**
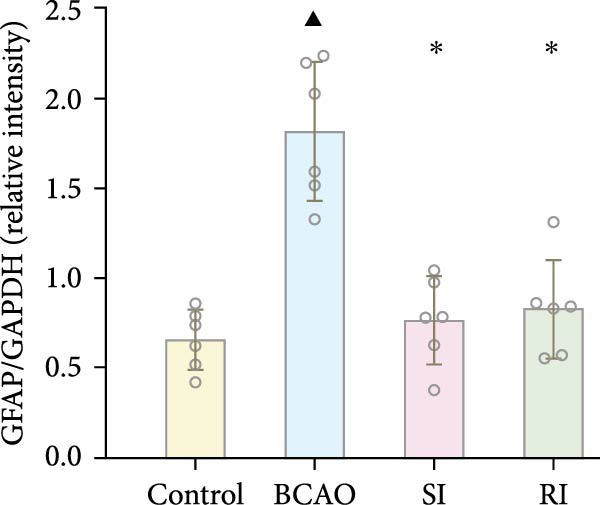
(B)

**Figure:**
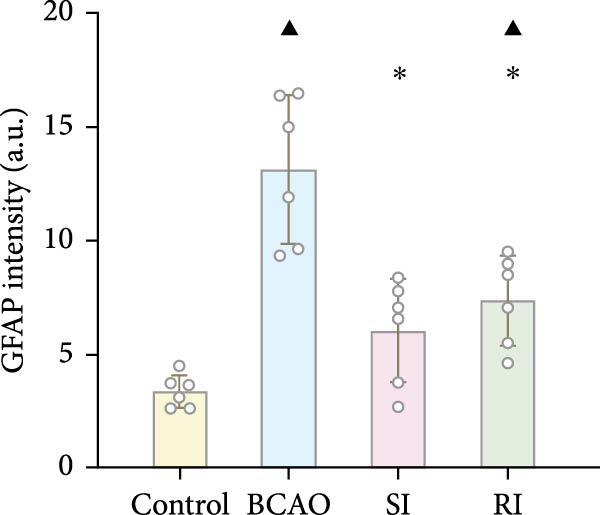
(C)

**Figure:**
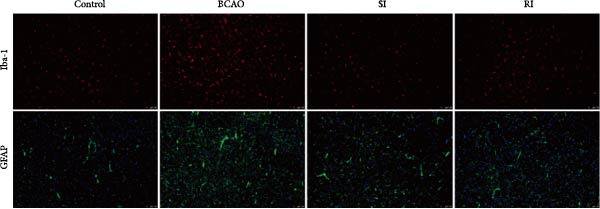
(D)

**Figure:**
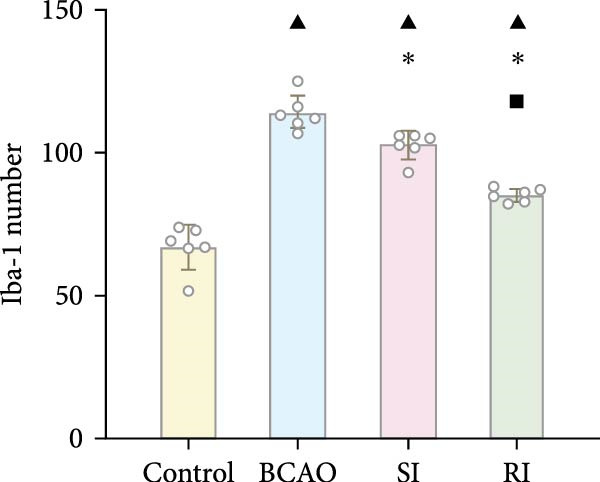
(E)

**Figure:**
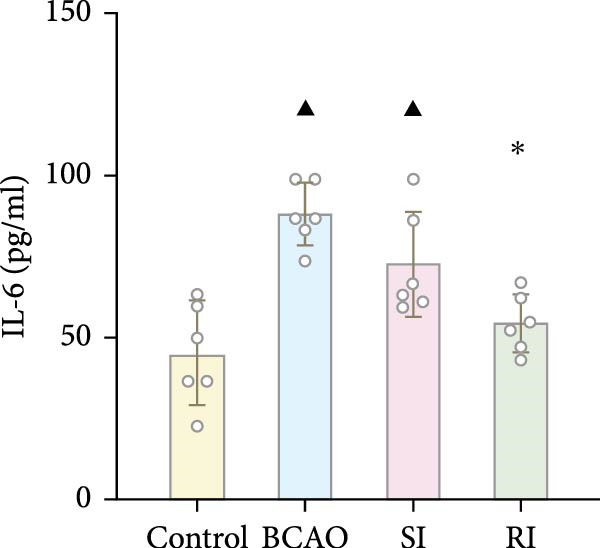
(F)

**Figure:**
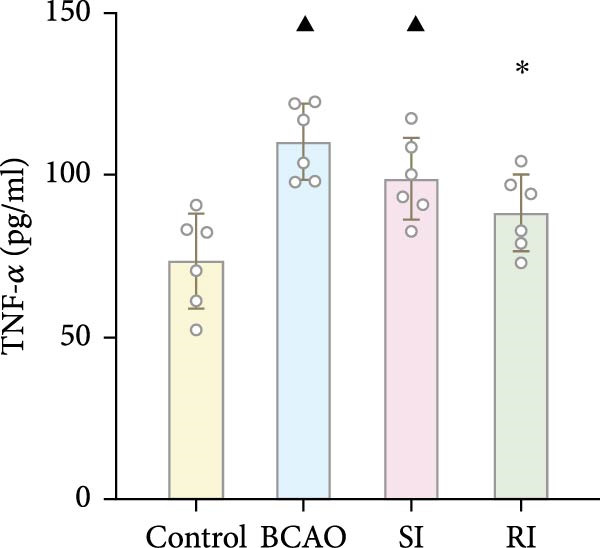
(G)

**Figure:**
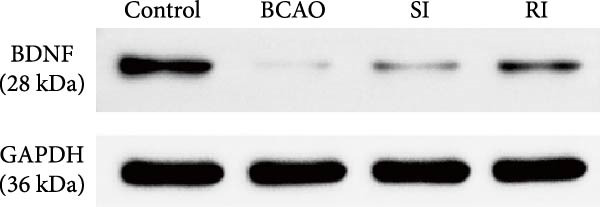
(H)

**Figure:**
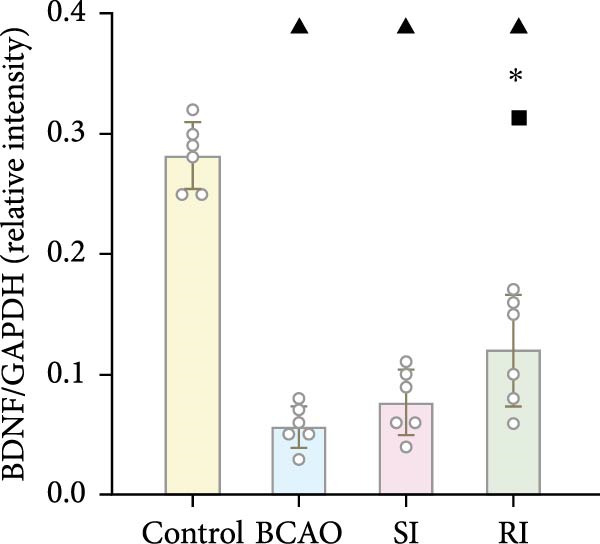
(I)

**Figure:**
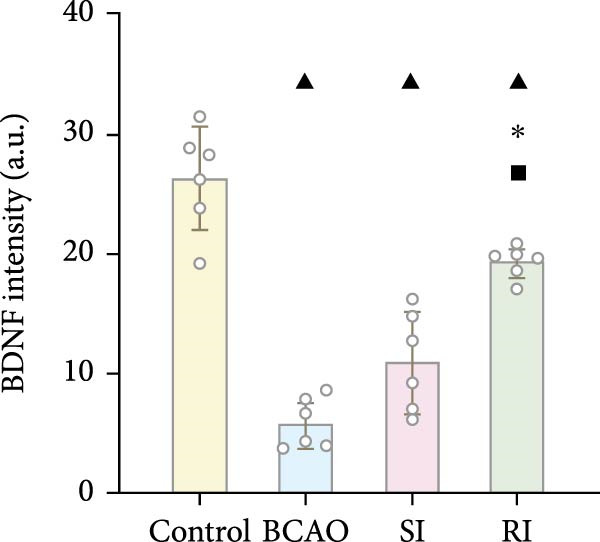
(J)

**Figure:**
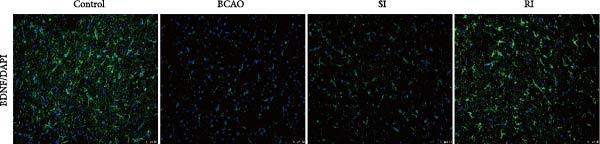
(K)

### 3.6. Repeated Injection of hUC‐MSCs Induced More Secretion of BDNF

To investigate the effect of single‐ and/or repeated‐dose hUC‐MSC administration on the neurotrophic effects of stem cells, we compared the expression levels of BDNF between the groups by WB and IF (Figure 5H,K, *p* < 0.01). BDNF expression in the Control group was significantly higher than in the other groups (Figure 5I,J, *p* < 0.01). The SI group showed improvement compared to the BCAO group, but the difference was not statistically significant. In contrast, the RI group showed a significant increase compared to the BCAO group (Figure 5I,J, *p* < 0.05), indicating that repeated injection had a significant effect on improving the neurotrophic environment of the central nervous system.

## 4. Discussion

Stem cell treatment is considered a promising approach for the treatment of moderate‐to‐severe CP [30]. However, stem cell treatment protocols still need to be thoroughly researched, which affects the efficacy [31]. In our previous research, we compared different stem cell transplantation routes in UCAO rats’ motor function and concluded that intrathecal administration may ultimately lead to the greatest benefit [25]. In this study, we found that repeated injection of hUC‐MSCs in BCAO rats could lead to significantly greater improvements in functional mobility and brain functioning compared with single injection. To our knowledge, this is the first study to use hUC‐MSCs in moderate‐to‐severe CP (BCAO model) and to compare the effects of single versus repeated intrathecal hUC‐MSC injections for CP.

### 4.1. Rationale for Model Selection: Addressing Translational Gaps in Preclinical CP Research

To our knowledge, no preclinical studies have specifically investigated hUC‐MSC therapy for moderate‐to‐severe CP. The widely used UCAO model has notable limitations [32, 33]: it induces only unilateral injury, inconsistent with the bilateral white matter damage typical in CP [34, 35]; it is performed at P7 in rats, missing the peak vulnerability period of pre‐oligodendrocytes (P2–P5) [36], which are central to the pathogenesis of immature white matter injury (WMI) [37, 38]; and it requires prolonged hypoxia (3 h), which exceeds most clinical hypoxic‐ischemic encephalopathy durations [39]. In contrast, the BCAO model better replicates the diffuse WMI and PVL common in children with CP [20]. Our results demonstrate that BCAO induces more severe functional impairment and neuropathological damage than UCAO, with MRI‐based RPSS grading confirming moderately severe injury. Thus, BCAO offers a more clinically relevant model for evaluating hUC‐MSC therapy in moderate‐to‐severe CP.

### 4.2. Rationale for Treatment Protocol and Mechanistic Insights

Repeated dosing was considered to reduce the risk of cell embolism caused by high‐dosage single administration [40]. The intervention window was strategically initiated at P21, corresponding to 2–3 years of human development—a period coinciding with peak myelination activity and representing an optimal therapeutic window for early intervention in CP [41]. Comparative assessment of single versus repeated intrathecal hUC‐MSC administrations revealed significantly enhanced therapeutic efficacy in the repeated dosing group. Rats receiving four injections between P21 and P49—spanning the critical period of neural plasticity in rats (equivalent to 2–18 years in humans)—demonstrated superior recovery of motor function and more substantial reduction in hypoxic‐ischemic neuronal damage [42]. This comprehensive coverage of the developmental plasticity window likely underlies the observed functional improvements. Our monitoring revealed no acute adverse effects from the repeated injection protocol. Concurrently, the gradual attenuation of the RFP fluorescence signal can be attributed to several possible mechanisms. Given the observed robust reduction in microglial activation (Iba‐1) and pro‐inflammatory cytokines (TNF‐*α*, IL‐6), immune‐mediated clearance by host microglia represents a plausible mechanism, which is consistent with the known phagocytic role of microglia and the transient nature of transplanted cells [43]. Other possibilities include proliferative dilution of the RFP label, cellular migration, or eventual apoptosis [44]. To clarify these dynamics, future studies will employ multi‐organ IF and high‐temporal‐resolution tracking to further elucidate the migration routes and long‐term fate of the transplanted cells.

### 4.3. Repeated hUC‐MSC Therapy Attenuates Neuroinflammation and Modulates Microglial Responses in CP

The processes of injury in CP could be divided into the transiently present acute phase, the secondary cellular response phase (involving cell death and metabolic disorders), and a long‐lasting third phase characterized by persistent inflammation [45]. Persistent inflammation may cause abnormal activation of neuroglial cells, which in turn could lead to ongoing brain damage, leaving CP patients susceptible to further functional impairment [46]. Microglia act as the “sentinel cells” of brain immunity [47]. After brain damage, microglia secrete larges amount of inflammatory factors such as TNF‐*α* and IL‐6, promoting their activation into M1 microglia, which then participate in the inflammatory cascade reaction and spread cell death beyond the initial ischemic area [48, 49]. When the inflammatory response is under control, M1 microglia transform into M2 microglia, which could downregulate inflammatory response factors and repair inflammatory damage [50, 51]. This study included microglial markers Iba‐1, TNF‐*α*, and IL‐6. We found that the levels of these inflammatory mediators in the SI group and RI group were lower than those in the BCAO group, and the downward trend in the RI group was more obvious. This suggests that repeated injection of hUC‐MSCs could better reduce the level of M1 microglia. At the same time, the level of neuronal marker NeuN increased, indicating that the neuronal survival rate improved after hUC‐MSC intervention, reflecting the repair of inflammatory damage. We hypothesized that the reduction in Iba‐1+ microglia after hUC‐MSC intervention was primarily due to a decrease in the number of M1 microglia, and that a small proportion of M1 microglia may polarize into M2 microglia to repair neuroinflammatory damage. However, due to the large reduction in M1 cells and the small number of M1 cells converted to M2, the number of microglia showed a downward trend overall. While the present study did not directly measure M2 markers, this mechanistic pathway is strongly supported by existing literature, which demonstrates that hUC‐MSCs can effectively promote M2 polarization [52–54]. Therefore, the observed anti‐inflammatory effects likely reflect a shift toward a reparative microglial phenotype.

### 4.4. Repeated hUC‐MSC Therapy Attenuates Reactive Astrogliosis and Promotes Myelin Regeneration

In addition to microglial activation, brain injury could also lead to reactive astrocyte proliferation. GFAP, a marker of reactive astrocytes, is upregulated during the formation of glial scars in the chronic phase of CP [20, 37–55]. Scar formation would inhibit axonal regeneration [56]. Myelin is an important component of neuronal structure. Restricted regeneration would affect the normal function of neural circuits, and thus affect motor function performance [57, 58]. In this study, both the RI and SI groups exhibited lower GFAP levels and increased myelin marker MBP. The degree of change was greater in the RI group than in the SI group, suggesting that stem cell intervention could reduce the number of reactive astrocytes and promote myelin regeneration. Considering that myelin regeneration is closely related to brain‐derived neurotrophic factors [59], this study also included BDNF. We found that only the BDNF content in the RI group showed significant differences compared with the BCAO group, which indirectly supports the conclusion that repeated stem cell injection is more effective in promoting myelin regeneration than a single injection.

## 5. Limitations

First, only short‐term cell tracking was performed, leaving the long‐term survival and distribution of transplanted cells after injection unassessed. Second, behavioral assessments were restricted to motor function, with no evaluation of cognitive or learning abilities. Third, while histological improvements were observed, no direct evidence confirmed neural differentiation or structural integration of hUC‐MSCs. Fourth, although mixed‐sex animal cohorts were used, outcomes were not analyzed by sex, despite known sexual dimorphism in neuroinflammatory responses [60, 61]. Finally, while the cell dose (1 × 10^6^) followed established regimens [62], a systematic dose–response analysis was not conducted.

## 6. Future Directions and Clinical Translation

To fully elucidate the therapeutic mechanisms of hUC‐MSCs in neural repair, future studies should integrate long‐term cell tracking with lineage tracing, expand behavioral assessments to include cognitive functions, incorporate sex‐stratified analyses, and conduct systematic dose‐response studies. At the cellular level, future studies should profile remyelination using oligodendrocyte markers (e.g., Olig2, NG2) and electron microscopy. Concurrent analysis of microglial polarization will elucidate immune modulation. Ultimately, these morphological and immune profiles must be correlated with electrophysiological and neuroimaging data to bridge cellular changes to functional circuit recovery.

The clinical translation of these findings faces several key challenges that must be addressed through collaborative multicenter efforts. These include comprehensive safety evaluation of repeated intrathecal injections, optimization of treatment protocols, development of predictive biomarkers for patient stratification, establishment of standardized manufacturing processes for clinical‐grade cells, and navigation of regulatory pathways for pediatric cell therapies. Successfully addressing these barriers will require systematically designed translational research programs that bridge preclinical findings with clinical application.

## 7. Conclusion

Our study showed that repeated injections of stem cells improved the motor function of rats with moderate‐to‐severe CP to a significantly greater extent than a single injection. We also confirmed that the BCAO model could simulate the motor dysfunction and brain injury observed in moderate‐to‐severe CP. In addition, we found that hUC‐MSCs could help repair white matter damage in the CC by promoting myelin regeneration. This improvement has the potential to enhance both cognitive and motor functioning in children with CP by improving information integration between brain hemispheres [63–65]. However, this study did not focus on the impact of hUC‐MSCs on cognitive function, which is worth further research.

**Nomenclature**
APC: Allophycocyanin
a.u.: absorbance unit
BCA: Bicinchoninic acid
BCAO: Bilateral carotid artery occlusion model
BDNF: Brain‐derived neurotrophic factor
BV421: Brilliant violet 421
CC: Corpus callosum
CD34: Hematopoietic progenitor cell antigen CD34
CD44: Cluster of differentiation 44
CD45: Protein tyrosine phosphatase receptor type C
CD73: Ecto‐5’‐Nucleotidase
CD105: Endoglin
CP: Cerebral palsy
DAPI: 4’,6‐diamidino‐2‐phenylindole
DTI: Diffusion tensor imaging
ELISA: Enzyme‐linked immunosorbent assay
FITC: Fluorescein isothiocyanate
g: Gram
GAPDH: Glyceraldehyde 3‐phosphate dehydrogenase
GFAP: Glial fibrillary acidic protein
hUC‐MSCs: Human umbilical cord‐derived mesenchymal stem cells
Iba‐1: Ionized calcium‐binding adapter molecule‐1
IF: Immunofluorescence
IL‐6: Interleukin‐6
M1: Primary motor cortex
MBP: Myelin basic protein
mL: Milliliter
MOI: Multiplicity of infection
MRI: Magnetic resonance imaging
MSCs: Mesenchymal stem cells
NeuN: Neuronal nuclear antigen
PE: Phycoerythrin
pg: picogram
pre‐OL: pre‐oligodendrocyte
PVL: Periventricular leukomalacia
P: Postnatal day
RFP: Red fluorescent protein
RI: Repeat injection
RPSS: Rat pup severity score
s: Second
SI: Single injection
TNF‐*α*: Tumor necrosis factor alpha
UCAO: Unilateral carotid artery occlusion model
WB: Western blot
WMI: White matter injury.

## Ethics Statement

The Experimental Animal Ethics Committee of Guangzhou Medical University approved all the experimental procedures (Acceptance Number: G2020‐257).

## Consent

The authors have nothing to report.

## Disclosure

All authors read and approved the final manuscript and the submission to this journal. This article is not published nor is under publication elsewhere. The funders played no role in the design, conduct, or reporting of this study.

## Conflicts of Interest

The authors declare no conflicts of interest.

## Author Contributions

Kaishou Xu contributed to conception and design, data analysis and interpretation, and provided financial support. Lu He contributed to conception and design, data analysis and interpretation. Yi Xu, Shiya Huang, and Xiaolin Guo contributed to experimental procedure. Liru Liu, Tingting Peng and Mingshan Han performed data curation, data analysis and interpretation. Ting‐Ting Peng and Yi Xu designed and prepared the figures. Ting‐Ting Peng, Yi Xu and Jie Luo participated in the writing. Ting Gao, Hongmei Tang and Jing Zhang contributed to supervision. Yi Xu, Ting‐Ting Peng, Shiya Huang and Xiaolin Guo should be listed as co‐first authors.

## Funding

The work was supported by the Featured Clinical Technique of Guangzhou (Grant 2023C‐TS59), the Guangzhou Municipal Science and Technology Project (Grant 2024A03J01274), the Major Innovation Technology Construction Project of Synergistic Chinese Medicine and Western Medicine of Guangzhou (Grant 2023‐2318), and the Plan on Enhancing Scientific Research in Guangzhou Medical University (Grant GMUCR2024‐02020).

## Data Availability

Further details and date of the study can be requested from the corresponding author.
